# 

**Published:** 1898-03

**Authors:** 


### CONTENTS:

SELECTIONS.

Article I.—Necrosis of Hard Palate. By Dr. H. C. Gilchrist...... 481

" II.—The Treatment of Teeth Having Putresent Pulps. By Dr. F. P. Hamley.... ........................ 483

" III.—Value of Examination of the Mouth in the Choice of of a Nurse. By Dr. V. Jarre........................ 487

" IV.—New Operative Method for the Radical and Rapid Cure of Chronic Empyemia of Maxillary Sinus..... 488

" V.—Shall the Dentist Advertise. By William W. Belcher, D. D. S., .......... .............. ............... 490

" VI.—The Adaptation and Retention of Artificial Dentures By A. O. Hunt, D. D. S.............................. 494

" VII.—The Fracture of Platinum Pins in Teeth and the Cracking of teeth in Soldering. By E. Duval...... 497

" VIII.—Treatment of Protrusion of the Lower Teeth. By Dr. J. N. Farrar........................................ 501

" IX.—Is the Dental Profession Crowded...................... 503

" X.—On Certain Difficulties of Diagnogis. By J. P. Parkinson, M. D., M. R. C. P.,.................................. 505

" XI.—Stimulants and Sedatives...... ....................... 518

" XII.—Saving an Old Tooth. By Ernest M'Caffey........... 520

" XIII.—Appendicitis. By C. Alvin Strasburg, M. D........... 524

MONTHLY SUMMARY.

The Care of Vulcanizers........................................ 525

Making Partial Dentures........................................ 526

For Burns.... .................................................. 527

Setting Bridges................................................ 528

Be Cleanly..................................................... 528

